# Adaptation of the Transition Readiness Assessment Questionnaire for Pediatric-Onset Multiple Sclerosis: Protocol for a User-Centered Design Approach

**DOI:** 10.2196/91763

**Published:** 2026-06-24

**Authors:** Claudia Gambrah-Lyles, Aaron W Abrams, Sara Loud, Hollie Schmidt, Emily Blosberg, Melissa Wright, Kelsey E Poisson, Kristen S Fisher, Kimberly A O'Neill, Akash Virupakshaiah, John W Rose

**Affiliations:** 1 Department of Neurology, School of Medicine, Washington University in St. Louis, 1 Children's Place, St. Louis, MO, 63110, United States, 1 314 471 2986; 2 Department of Neurology, Center for Pediatric Neurosciences and Mellen Center for MS Treatment and Research, Cleveland Clinic Neurological Institute, Cleveland, OH, United States; 3 Accelerated Cure Project for Multiple Sclerosis, Waltham, MA, United States; 4 Mr Oscar Monkey, Elk Mound, WI, United States; 5 Department of Neurology, Division of Pediatric Neurology, University of Utah School of Medicine, Salt Lake City, UT, United States; 6 Department of Pediatrics, Division of Neurology, Nationwide Children’s Hospital, Columbus, OH, United States; 7 Department of Pediatrics, Division of Neurology and Developmental Neuroscience, Baylor College of Medicine and Texas Children’s Hospital, Houston, TX, United States; 8 Department of Neurology, NYU Grossman School of Medicine, New York, NY, United States; 9 Department of Neurology, Divisions of Pediatric Neurology and Neuroimmunology & Glial Biology, Weill Institute for Neurosciences University of California, San Francisco, San Francisco, CA, United States; 10 Department of Neurology, University of Utah School of Medicine, Salt Lake City, UT, United States

**Keywords:** adaptation, participatory research, transition to adult care, pediatric-onset multiple sclerosis, multiple sclerosis, implementation science

## Abstract

**Background:**

Pediatric-onset multiple sclerosis (POMS) is a chronic, progressive neurologic condition requiring lifelong management and coordinated transition from pediatric to adult care. Evidence-based guidelines identify transition readiness assessment as a core component of successful transition; however, most POMS clinics do not formally assess readiness, and existing tools do not address POMS-specific challenges, such as fluctuating disability, complex treatment regimens, and cognitive impairment. This gap underscores the need for a transition readiness measure tailored to POMS.

**Objective:**

This paper aims to describe a stakeholder-engaged, implementation science–guided protocol for adapting the Transition Readiness Assessment Questionnaire (TRAQ) version 6.0 to reflect the unique developmental and clinical needs of youth with POMS.

**Methods:**

Using adaptation and participatory research as our guiding implementation strategies, surveys will be administered to patients, caregivers, and clinicians to identify barriers to and facilitators of transition to adult care and define essential self-management competencies in POMS. Survey content will be informed by constructs from the dynamic adaptation process framework and existing TRAQ 6.0 domains. Identified competencies will be refined using a Delphi consensus process. Through cognitive interviewing and a multidisciplinary focus group of collaborators, the adapted measure will be reviewed to assess clarity, relevance, and perceived clinical utility.

**Results:**

This project will generate a consensus-driven set of POMS-specific transition competencies and systematically adapt the TRAQ 6.0 to the POMS population. This study was funded in July 2025. The health care provider survey was disseminated in November 2025 across the 27 sites in the US Network of Pediatric Multiple Sclerosis Centers. The patient and caregiver surveys have been finalized, and distribution is projected to begin in June 2026, with a target of 15 patients and 15 caregivers. As of manuscript submission, no patients or caregivers have been recruited, and data analysis has not yet started. Data collection is expected to conclude by the end of 2026, and results are anticipated for publication in 2027.

**Conclusions:**

This protocol outlines a rigorous, replicable approach to adapting a validated transition readiness measure to POMS. The adapted TRAQ 6.0 will support evidence-based transition planning and inform future psychometric testing and implementation research to improve the care of patients with POMS as they age.

## Introduction

### Background

An estimated 2.8 million people worldwide have multiple sclerosis (MS) [1], a chronic central nervous system disease that causes debilitating neurological impairment and requires lifelong treatment and care. Pediatric-onset MS (POMS) accounts for up to 10% of cases [2-4], with incidence and prevalence continuing to rise [5-8]. Compared to adult MS, POMS demonstrates aggressive onset [9], high relapse rates [10], and early disability [8,9,11-13]. Similar to other chronic diseases of childhood, effective management of POMS relies on a smooth transition to adult care. However, research on transition to adult care in POMS and strategies to support the process remain scarce [14-18].

The Six Core Elements of Health Care Transition provide an evidence-based framework for transition planning [19-23]. A core strategy in the Six Core Elements is transition readiness assessment, which evaluates a young patient’s preparedness for taking on adult health care responsibilities. Higher transition readiness scores are associated with improved self-management skills and reduced disease-related complications during transition to adult care [24-27]. Furthermore, implementing the Six Core Elements into clinical practice has demonstrated benefits, including improved clinical outcomes, treatment adherence, and patient satisfaction [20,28-30].

Despite these benefits, preliminary survey data from 10 major POMS clinics in the United States reveal variable transition to adult care practices and lack of adherence to the recommendations of the Six Core Elements—most notably, 80% do not formally assess or track transition readiness. These gaps are also reflected in the literature, revealing that patients with POMS feel unprepared for the transition to adult care process [27-31] and experience poor medication adherence and increased disability after leaving pediatric-focused care [31]. This disconnect among guidelines, clinical practice, and patient experience highlights a critical need to develop strategies to support implementation of evidence-based transition to adult care practices in POMS.

Although transition readiness tools have been adapted for other pediatric conditions [32-36], no measure captures the specific challenges that patients with POMS experience, including lifelong immunomodulation and immunosuppression, fluctuating disability and unanticipated relapse, and disease progression in the absence of relapse activity [16,37-44].

This work is grounded in the premise that variability and low adoption of evidence-based practices often arise from a misalignment between interventions and the needs, values, or workflows of the populations and settings they are intended to serve [45-47]. Without a tailored transition readiness tool, patients with POMS may face increased risk of poor disease self-management, disengagement from MS-targeted care, and worse long-term outcomes upon transition to adult-based care because current transition tools do not adequately capture their unique clinical and self-management needs.

### Scale Development and Adaptation in Implementation Science

Adaptation is a cornerstone of implementation science that directly influences the adoption, sustainability, and clinical impact of an intervention. The Expert Recommendations for Implementing Change highlight “promoting adaptability” as a key strategy [48]. While frameworks such as the dynamic adaptation process (DAP) [49] and the discover, design/build, and test (DDBT) [50] model provide clear road maps for adapting interventions, systematic guidance for adapting clinical measures remains less explicit.

This project aims to bridge this gap by applying intervention-focused adaptation frameworks to a transition readiness measure. Rather than developing an instrument de novo, we build on the Transition Readiness Assessment Questionnaire (TRAQ) version 6.0, leveraging its established psychometric validity [51] and extensive prior adaptation [32,34,52-57]. Our approach integrates DAP constructs to balance fidelity to the original tool while integrating the POMS-specific context using DDBT principles, which prioritize iterative stakeholder feedback. By treating measure adaptation as a systematic, stakeholder-engaged process, we aim to produce a POMS-specific tool optimized for real-world clinical adoption and usability.

## Methods

### Overall Study Design and Framework

This study applies an integrated DAP and DDBT framework to adapt the TRAQ 6.0 to POMS. We focus specifically on the item development and refinement phase, where contextual determinants and stakeholder input are most critical.

The DAP guides the multilevel needs assessment by identifying system, health care provider, and patient and caregiver determinants to signal where adaptation is necessary. It provides a structure for balancing fidelity to the original TRAQ 6.0 with modifications that address POMS-specific barriers, facilitators, and readiness factors. The DDBT framework structures the user-engaged adaptation process. A multidisciplinary team synthesizes targeted feedback from patients, caregivers, and clinicians to iteratively refine item wording and domain definitions. This process ensures that revisions directly reflect user priorities and support clinical feasibility.

### Participants

Effective adaptation requires active involvement from all key partners—patients, clinicians, and community members; to achieve this, we will leverage our robust, existing collaborations. Health care provider and clinician perspectives will be obtained from the US Network of Pediatric Multiple Sclerosis Centers (NPMSC), a national network of clinical sites caring for patients with POMS, and a data coordinating and analysis center dedicated to advancing POMS care [54].

To capture patient and caregiver perspectives, we will partner with iConquerMS, a community-based consortium and patient-powered research network with over 7500 members. In further alignment with this study, iConquerMS has dedicated platforms for patients with POMS and their caregivers, including Mr Oscar Monkey (a nonprofit organization for youth MS), for advancing POMS-specific research initiatives.

**Figure 1.**
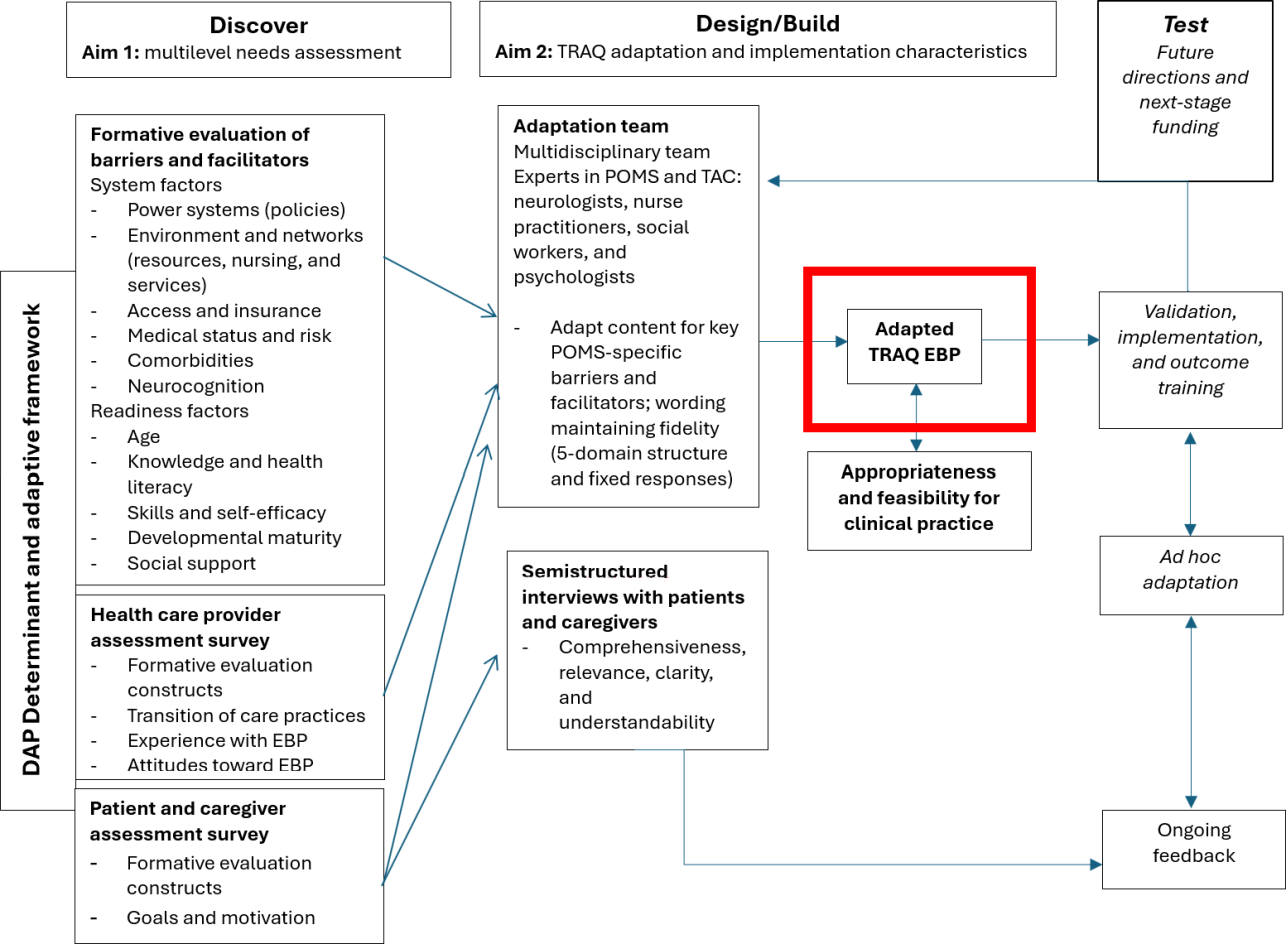
Integrated dynamic adaptation process (DAP) and discover, design/build, and test framework demonstrating the process of creating an adapted Transition Readiness Assessment Questionnaire (TRAQ) for the pediatric-onset multiple sclerosis (POMS)–specific context. EBP: evidence-based practice; TAC: transition to adult care.

As illustrated in Figure 1, the study moves from a needs assessment (“discover”) to item and domain refinement with implementation considerations (“design/build”) and, ultimately, positions the adapted measure for future validation and implementation testing (“test”). This design is operationalized through surveys completed by health care providers, patients, and caregivers to capture these determinants systematically and a Delphi process to guide the adaptation, followed by cognitive and semistructured interviews to assess the clarity, relevance, completeness, and clinical utility of the adapted tool.

### Discover Phase: Survey-Based Multilevel Needs Assessment

The health care provider, patient, and caregiver surveys draw on constructs from the DAP and domains from the TRAQ 6.0. The goal of this exploratory phase is to identify context-specific needs and priorities to guide item modification.

The health care provider survey will assess current transition to adult care practices, barriers and facilitators, and health care provider perspectives on essential transition readiness skills. Health care providers will be recruited from the US NPMSC, which includes 27 participating sites across the United States. Eligible participants include the principal physician health care provider at each site. All US NPMSC sites are invited to participate; representativeness across hospital type (freestanding children’s hospital, children’s hospital within a larger system, and adult hospital with a pediatric unit), geographic location, practice setting (academic vs community), estimated number of patients with POMS, and health care provider training will be assessed descriptively.

The patient and caregiver surveys will examine perceived knowledge gaps, critical transition skills, and key challenges. Likert-scale items will assess the perceived importance of domains such as disease understanding, medication management, and appointment scheduling. Questions related to MS self-management were drawn from previously published tools for evaluating skills and knowledge in MS. In addition, prior studies have reported that self-management skills that youth with POMS want to develop during transition include diagnosis disclosure and concealment, creating safety nets, and setting expectations [44,58].

Surveys will target patients with POMS aged 14 to 26 years and their caregivers to capture experiences across transition stages. The target sample for survey completion is approximately 15 patients and 15 caregivers. Eligible patients must have a confirmed POMS diagnosis (symptom onset before the age of 18 years) and currently participate in iConquerMS Kids & Teens or Mr Oscar Monkey. Eligible caregivers must be the parent or legal guardian of a current or former patient with POMS who meets the age eligibility criteria. Participants will be excluded if they are unable to complete the survey in English.

Given the rarity of POMS, we will use convenience sampling through iConquerMS and its dedicated youth MS platforms to reach a sufficient number of patients and caregivers.

### Design/Build Phase: TRAQ 6.0 Adaptation

The TRAQ 6.0 includes 20 items across 5 domains essential to transition readiness—managing medications, appointment keeping, tracking health issues, communicating with health care providers, and managing daily activities. These domains serve as the structural base for adaptation, allowing POMS-specific content to be integrated without altering the underlying organization of the measure.

Survey data from health care providers, patients, and caregivers will be used to identify POMS-specific challenges, skill gaps, and contextual factors not captured in the current TRAQ 6.0. This process will focus on integrating POMS-specific needs—such as managing fatigue, cognitive changes, diagnosis disclosure decisions, and complex treatment regimens while preserving the TRAQ’s existing domain framework. Prior studies have shown that youth with POMS have identified these areas as priorities for self-management during transition, yet they are not meaningfully captured in any currently available transition readiness or self-management measure, including the TRAQ [44,58]. These findings will generate an expanded list of potential competencies relevant to transition readiness in POMS.

A Delphi process will then be used to refine and prioritize these competencies. A multidisciplinary panel will rate each competency on importance, relevance, and fit within the TRAQ 6.0 domain structure. The panel will include 8 to 10 members representing clinicians, patients, and caregivers, all considered voting members. Clinicians on the panel will be drawn from the NPMSC Transition to Adult Care Working Group. Patients and caregivers on the Delphi panel will be recruited from iConquerMS and selected based on prior involvement in POMS advocacy and research leadership, such as board members of Mr Oscar Monkey.

Competencies will be rated on a 5-point Likert scale for importance and relevance. Consensus will be defined as more than 70% of panelists rating a competency as “important” or “very important” (Likert score of 4 or 5). A minimum of 2 and a maximum of 3 rounds are planned. After each round, panelists will receive anonymous, aggregated results and may revise their ratings in light of group feedback. Items not reaching consensus by the final round will be reviewed by the core study team (the authors of this publication). The team will draw on panelist free-text comments from each round to inform a final decision to retain, modify, or exclude the item, and the reasoning for each decision will be recorded in the adaptation tracking log maintained by the core study team. Across successive rounds, controlled feedback will promote consensus on which competencies are essential and should be represented in the adapted measure.

Once the Delphi process is complete, these competencies will be mapped onto the TRAQ using the following set of adaptation decision rules to convert the finalized Delphi competencies into revised or new TRAQ 6.0 items: (1) existing TRAQ items that adequately capture a Delphi-endorsed competency will be retained without modification, (2) existing items that partially capture a competency but require language changes to reflect POMS-specific content (such as references to MS medications, relapse recognition, or disease-modifying therapy) will be reworded, (3) Delphi-endorsed competencies not represented in existing TRAQ items will be developed as new items, and (4) existing TRAQ items that do not correspond to any Delphi-endorsed competency or that are redundant for the POMS population will be removed or merged.

### Test Phase: Refinement

Finally, cognitive interviews will be conducted in 2 iterative rounds with 3 to 5 patients and 3 to 5 caregivers recruited through iConquerMS. Each round will evaluate item clarity, comprehensiveness, and relevance, with findings used to refine item wording, content, and response options before the next round. The sample size is consistent with established recommendations for cognitive interviewing in rare disease populations.

Following completion of both rounds, a multidisciplinary focus group of 8 to 10 clinicians, patients, and caregivers (none of whom participated in the cognitive interviews or Delphi process) from the US NPMSC and iConquerMS will assess the adapted measures’ overall clinical feasibility, comprehensiveness, and suitability for routine use in POMS care via brief semistructured interviews.

Subsequent phases (outside the scope of this study) will include psychometric testing, domain confirmation, and broader implementation evaluation.

### Ethical Considerations

This study was reviewed and approved by the Washington University in St. Louis Institutional Review Board (IRB; 202508024). All study procedures will be conducted electronically. Health care provider participation was granted a waiver of informed consent by the IRB. Electronic informed consent will be obtained from adult patients (aged 18 years and older) and caregivers prior to participation. For adolescent participants under the age of 18 years, electronic parental or guardian consent and participant assent will be obtained in accordance with institutional and federal guidelines. All data will be collected and stored on REDCap (Research Electronic Data Capture; Vanderbilt University), a secure, HIPAA (Health Insurance Portability and Accountability Act)–compliant platform, and deidentified prior to analysis. All participants will be compensated for their time in accordance with the approved IRB protocol.

### Analysis

#### Quantitative Analysis

Survey data from health care providers, patients, and caregivers will be analyzed using descriptive statistics to characterize transition practices, perceived barriers and facilitators, and ratings of POMS-specific transition competencies. Mean scores, frequency distributions, and 95% CIs will summarize item-level responses. Competency ratings will be aggregated to identify high-priority areas for inclusion in the Delphi process. Group comparisons (eg, patient vs caregiver perspectives) will be explored using 1-tailed *t* tests or nonparametric equivalents as appropriate; these analyses are exploratory and intended to inform adaptation rather than test hypotheses.

#### Qualitative Analysis

Semistructured interview transcripts will undergo thematic analysis. Two independent coders will develop a shared codebook using an inductive-deductive approach informed by DAP constructs and TRAQ 6.0 domains. Themes will be used to refine competency definitions, identify gaps not captured in the quantitative data, and guide item wording during adaptation workshops. Discrepancies will be resolved through consensus.

#### Sample Size Considerations

The study is designed for concept and item development rather than psychometric estimation; therefore, sample adequacy will be determined by the need for representation across stakeholder groups rather than statistical power. The health care provider survey targets all principal health care providers across the 27 US NPMSC sites. The patient and caregiver surveys will each target 15 patients and 15 caregivers recruited through iConquerMS across a broad age range and at various transition stages to ensure diversity of experience.

The Delphi panel will consist of 8 to 10 experts representing clinicians, patients, and caregivers, consistent with established standards for consensus development [59]. For cognitive interviews, 2 iterative rounds of 3 to 5 patients and 3 to 5 caregivers will be conducted, consistent with established recommendations for cognitive interviewing [60]. The final focus group will include 8 to 10 clinicians, patients, and caregivers recruited from the US NPMSC and iConquerMS.

## Results

The project was funded in July 2025. The health care provider survey was disseminated in November 2025 across the 27 sites in the US NPMSC. The patient and caregiver surveys have been finalized, and distribution is projected to begin in June 2026, with a target of 15 patients and 15 caregivers. As of manuscript submission, no patients or caregivers have been recruited, and data analysis has not yet started. Data collection is expected to conclude by the end of 2026, with results anticipated for publication in 2027.

## Discussion

### Expected Findings

This study will produce the first transition readiness assessment tailored specifically to POMS. We anticipate that the adapted TRAQ will extend beyond the original domains to reflect competencies unique to this population, including managing immunomodulatory therapies, relapse recognition and reporting, cognitive and fatigue-related challenges, and engagement with specialist-driven adult MS care. Addressing these gaps has direct clinical relevance as it will allow for a standardized means of assessing and tracking transition readiness. A POMS-specific tool has the potential to strengthen preparation for adult-focused care, improve self-management skills, and promote continuity across the transition period. Future work will evaluate the extent to which interventions and strategies informed by this measure translate into improved disease-related outcomes and quality of life.

Furthermore, a key contribution of this work is the application of intervention adaptation frameworks to an existing measure. By integrating constructs from the DAP and the DDBT model, the study offers a transparent, easily replicable approach to measure adaptation grounded in implementation science. The combined use of surveys, consensus methods, and user-centered qualitative feedback will ensure that fidelity to a validated instrument is balanced with context-specific modifications in response to patient, caregiver, and clinician needs.

This framework-guided approach provides a model for adapting other clinical measures and positions the adapted TRAQ 6.0 for future psychometric testing and broader implementation in POMS. Comparison to prior adaptation work further underscores the importance of this protocol. Existing TRAQ adaptations have targeted disease-specific needs in conditions such as epilepsy [32], spina bifida [34], and sickle cell disease [33,35,36]. While these efforts have advanced the field of transition measurement, none has addressed the distinct clinical profile of POMS. A systematic review of self-management outcome measurement instruments found that all versions of the TRAQ demonstrated sufficient content validity only for general youth with chronic diseases, with content-linking analyses revealing systematic gaps in domains most relevant to youth with MS—including social reputation, cognition, fatigue, and attitudes toward their health condition [57]. Qualitative work with transitioning youth with MS further confirmed that these domains are self-management priorities not captured by existing tools [58]. Our protocol directly addresses these evidence-based gaps, applying the DAP and DDBT frameworks to generate a POMS-specific adapted measure that also incorporates dedicated stakeholder engagement.

### Strengths and Limitations

Key strengths of this protocol include the engagement of a nationally distributed, established clinical network (US NPMSC) and a patient-powered research network (iConquerMS) with dedicated POMS infrastructure, enabling access to a rare population that would be difficult to recruit through single-site efforts. The multi-stakeholder design, including patients and caregivers as Delphi voting members, reflects a genuine commitment to co-design and is consistent with best practices in implementation science. Limitations include the relatively modest sample sizes planned for the patient and caregiver surveys, which reflect the rarity of POMS and practical recruitment constraints; while adequate for content and item development, they may limit generalizability to the broader POMS population. Additionally, as all participating sites for the health care provider survey are US NPMSC members—predominantly academic centers—generalizability to community-based settings or non-US health care contexts may be limited. Future work should evaluate the adapted TRAQ in community and international POMS samples.

### Clinical Implementation

The adapted TRAQ is intended for use in routine POMS clinical encounters as part of structured transition planning. It can be administered directly to patients and/or caregivers, ideally introduced during annual or transition-focused visits beginning at the age of 14 to 16 years. Clinicians and care coordinators can use the completed measure to identify skill gaps and guide individualized transition support. The instrument will be suitable for both paper-based and electronic administration via platforms such as REDCap or electronic health record patient portals. Future implementation phases will evaluate feasibility, acceptability, and integration into clinical workflows across NPMSC sites.

### Dissemination

The results of this study will be disseminated through peer-reviewed publications, conference presentations at national neurology and transition medicine meetings, and direct engagement with the US NPMSC and iConquerMS communities. The adapted TRAQ, once psychometrically validated, will be made openly available to clinicians and researchers to support adoption across POMS and broader pediatric neurology settings.

## Abbreviations

DAP: dynamic adaptation process
DDBT: discover, design/build, and test
HIPAA: Health Insurance Portability and Accountability Act
IRB: institutional review board
MS: multiple sclerosis
NPMSC: Network of Pediatric Multiple Sclerosis Centers
POMS: pediatric-onset multiple sclerosis
REDCap: Research Electronic Data Capture
TRAQ: Transition Readiness Assessment Questionnaire
